# An Application of Evolutionary Game Theory to Social Dilemmas: The Traveler's Dilemma and the Minimum Effort Coordination Game

**DOI:** 10.1371/journal.pone.0093988

**Published:** 2014-04-07

**Authors:** Swami Iyer, Joshua Reyes, Timothy Killingback

**Affiliations:** 1 Computer Science Department, University of Massachusetts, Boston, Massachusetts, United States of America; 2 Department of Systems Biology, Harvard Medical School, Boston, Massachusetts, United States of America; 3 Mathematics Department, University of Massachusetts, Boston, Massachusetts, United States of America; University of Maribor, Slovenia

## Abstract

The Traveler's Dilemma game and the Minimum Effort Coordination game are two social dilemmas that have attracted considerable attention due to the fact that the predictions of classical game theory are at odds with the results found when the games are studied experimentally. Moreover, a direct application of deterministic evolutionary game theory, as embodied in the replicator dynamics, to these games does not explain the observed behavior. In this work, we formulate natural variants of these two games as smoothed continuous-strategy games. We study the evolutionary dynamics of these continuous-strategy games, both analytically and through agent-based simulations, and show that the behavior predicted theoretically is in accord with that observed experimentally. Thus, these variants of the Traveler's Dilemma and the Minimum Effort Coordination games provide a simple resolution of the paradoxical behavior associated with the original games.

## Introduction

Social dilemmas embody the tension between individual self-interest and the common good that is inherent in many important situations in the real world. In a social dilemma, individually reasonable behavior results in a situation in which all individuals are less well off than they could otherwise have been [1]. Social dilemmas underlie many of the most fundamental and intractable problems in the biological and social sciences, such as the evolution of cooperation [2] and the efficient use of limited shared resources [3]. From a more formal point of view, a social dilemma can be modeled as a game in which there exists at least one Hicks inefficient Nash equilibrium. It is Hicks inefficient (i.e. socially inefficient) in that there is at least one other outcome in which all individuals would be better off, and since it is a Nash equilibrium there is no incentive for any individual to change their behavior [1]. Examples of 2-person games that are social dilemmas include: The Prisoner's Dilemma [2], [4], [5], the Snowdrift game (also known as the Chicken or Hawk-Dove game) [6], and the Stag-Hunt game (also known as the Assurance game) [7]. Multi-person social dilemmas include, the Public Goods game [8], [9] and the Tragedy of the Commons [3]. While the original game theory formalizations of these social dilemmas typically involved discrete-strategy games, more recently continuous-strategy versions of the Prisoner's Dilemma game [10], the Snowdrift game [11], the Tragedy of the Commons [12] game, and the Public Goods game [13] have been formulated and studied.

One particularly fascinating class of social dilemmas are those for which the predictions of game theory appear to be inconsistent with the behavior observed when the games are played experimentally [14]. The Traveler's Dilemma (TD) game [15]–[17] and the Minimum Effort Coordination (MEC) game [17], [18] are two celebrated examples of such games.

These games challenge the notion that the rational solution to a game, as embodied in the concept of the Nash equilibrium, accurately describes the behavior of humans engaged in these social dilemmas. In the TD game there exists a unique, pure strategy, Nash equilibrium that is undesirable for all concerned. In contrast to the TD game, in the MEC game every pure strategy is a Nash equilibrium, and thus the rational solution concept lacks any prescriptive or predictive power.

**Figure 1:**
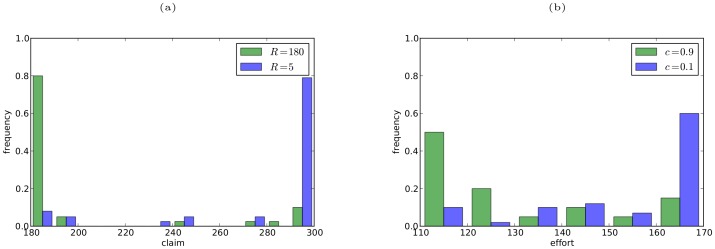
Results from playing the TD and MEC games with human subjects, adapted from [17]. (a) TD game: individuals make higher claims when the reward/punishment parameter 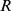 is low, and make lower claims when 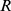 is high. (b) MEC game: individuals expend more effort when the effort cost 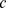 is low, and less effort when 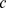 is high.

The TD game is conventionally introduced through a story of the following form. Two travelers, on their return journey from an exotic country, find that their luggage containing identical souvenirs has been lost by the airline. The officer in the claims department puts them in separate rooms, hands each of them a claims form, and tells them that they can claim any integer amount between 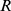 and 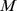 (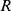 and 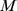 are assumed to be positive integers with 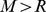). He also informs them that if they both ask for the same amount, they will be paid that amount, and if they ask for different amounts, each will be reimbursed at the lower value, but with a penalty 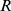 deducted from the higher claimant (who is assumed to have lied) and given to the lower claimant (as a reward for being honest). Thus, the TD game is a 2-person game with the discrete strategy set 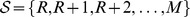, and payoff to an 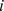-claimant against a 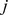-claimant is defined by 
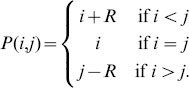
(1)In this context a Nash equilibrium is a pair of claims, such that, if each claim is known to the other traveler then neither has reason to revise their claim. For 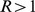, there is an incentive for each traveler to undercut any common claim. Using backward induction, it is not hard to see that the travelers should each claim the amount 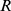, i.e., 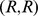 is a unique Nash equilibrium for the TD game. Thus, the unique Nash equilibrium of the TD game is the paradoxical outcome in which both travelers claim the lowest possible amount. We note, in particular, that the Nash equilibrium is independent of the reward/punishment parameter 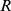. However, as intuition would suggest, this is not how individuals actually play this game [17], [19]–[22]. For instance, [22] found the following results when they played the game with 50 subjects (25 pairs). The subjects could make claims between 180 and 300, in two treatments, one with 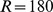 and another with 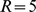. The results are shown in Figure 1(a). In the high-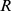 treatment, close to 80 percent of all the subjects chose the Nash equilibrium strategy, with an average claim of 201. However, in the low-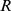 treatment, roughly the same fraction chose the highest possible claim, with an average value of 280. Since the unique Nash equilibrium prediction is independent of the parameter 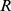, classical game theory is unable to explain the most salient feature of these experimental results, namely, the effect of the reward/punishment parameter 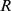 on average claim levels.

The MEC game has a somewhat similar flavor to the TD game, in that the payoffs are again determined by the minimum of two actions [17], [18], [20]. In this game the players choose integer effort levels between 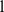 and 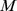 (where 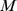 is assumed to be an integer greater than 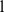), and a player's payoff is given by the minimum of the two effort levels minus the cost of the player's own effort. Therefore, the MEC game is a 2-person game with the discrete strategy set 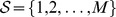, and payoff to an 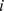-strategist against a 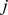-strategist defined by 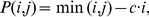
(2)where 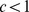 is a cost parameter. The MEC game suffers from the opposite problem to that of the TD game. Instead of exhibiting a single, deficient, Nash equilibrium, the MEC game exhibits multiple Nash equilibria; it is easy to see that any common effort level is a Nash equilibrium. Moreover, standard refinements of the Nash equilibrium concept do not select a subset of the equilibria. For instance, the Nash equilibria are strict, and thus trembling hand-perfect. Hence, classical game theory provides no obvious criterion to choose among them.

As with the TD game, when the MEC game is actually played with human subjects the observed behavior is inconsistent with the results predicted by game theory [17], [18]. For example, [17] found the following results in their experiment. The subjects could chose integer effort levels from 110 to 170, in one of two treatments, a low effort cost treatment of 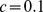 and a high effort cost treatment of 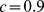. The results are shown in Figure 1(b). In the low effort cost treatment the behavior is concentrated close to the highest effort level of 170, while in the high effort cost treatment the preponderance of the effort levels are at the lowest possible value. These results clearly indicate that the effort levels employed by subjects are inversely related to the effort costs, despite the fact that any common effort level is a Nash equilibrium.

The paradoxical results obtained for the TD and MEC games using classical game theory are not resolved by instead using standard deterministic evolutionary game theory. Since the unique Nash equilibrium 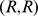 in the TD game is strict it is a globally stable equilibrium point for the replicator dynamics [23]. Hence, the replicator dynamics of the TD game will always converge to the minimum claim level 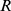. Similarly, in the MEC game every common effort level is a strict Nash equilibrium and hence a stable equilibrium point for the replicator equations. Thus, the behavior of the replicator dynamics does not select any subset of the Nash equilibria. The paradoxical nature of both games is, therefore, equally evident when studied using either classical game theory or deterministic evolutionary game theory.

It is noteworthy that the importance of the TD game and the MEC game are rather similar in nature. The TD game is theoretically significant because it exposes so clearly an apparently paradoxical aspect of game theory: namely the inability of the Nash equilibrium concept to predict the actual behavior of individuals interacting in this type of game. Moreover, it has been observed in [24] that the TD models competitive egg ejection in a species of communally nesting birds, the Greater Ani [25], [26]. In this species, if two females share a nest then each female chooses a time to change from ejecting eggs from the nest to laying eggs. If both select an early time (which corresponds to a large claim), then both obtain a large payoff since they can both successfully lay many eggs. If, however, one chooses to wait, then she can eject the other birds already-laid eggs and obtain an even greater payoff, while at the same time inflicting a loss on the other bird [24]. Thus, this situation has the structure of the TD game.

The MEC game also has both theoretical and practical importance. It is theoretically significant because it starkly illustrates the lack of prescriptive or predictive power inherent in the notion of Nash equilibrium. In addition, it has considerable practical import since many interesting and important real-world situations can be modeled by MEC games [27].

As an example of how MEC games naturally arise let us consider two companies, denoted by A and B, each of which manufacture a critical component of a jointly produced product. Let us suppose that company A makes widgets, while company B makes grommets. The final product, containing both a widget and a grommet, is sold jointly, with the revenues being equally split between the two companies. Each company can choose the amount of effort to expend in producing its component, with higher effort levels resulting in components of higher quality. We shall assume that the performance of the final product, and thus also the revenues obtained from sales of the product, is limited by whichever of the two components has the lower quality. Therefore, the profits obtained by a given company from sales of the product may be expressed as the minimum of the efforts expended by each company to produce widgets or grommets, respectively, minus the cost associated with the companies' own effort. Thus, such a situation can be modeled by a MEC game.

The importance of the TD and MEC games, both theoretically and in practice, is clear from the above comments. Therefore, obtaining a satisfactory understanding of the dynamics of these games is of considerable significance, and it is the purpose of this paper to contribute to such an understanding.

A number of different theoretical approaches have been investigated as possible explanations of the behavior found empirically in the TD and MEC games. In one approach, stochastic learning models [17], [20], [28] have been proposed to explain the anomalous behavior observed in the TD and MEC games. A quite different approach, using stochastic evolutionary dynamics in finite populations [24], has also been investigated as a means of resolving the paradoxical features of the TD game. Other theoretical approaches to explaining the behavior of the TD game have been studied in [29]–[32]. Other approaches which explore how errors in a game may lead to deviations from Nash equilibrium play include [33]–[37].

Here we propose an alternative, considerably simpler, theoretical framework to explain the evolutionary dynamics of both the TD and MEC games, which accounts for the empirically observed behavior. Our method applies to a wide class of games that includes both the TD and MEC games. We first observe that while the TD and MEC games were originally formulated as discrete strategy games it is natural to consider variants of these games in which the strategies are continuously variable. In this paper we define these continuous-strategy variants of the TD and MEC games, which suffer from the same paradoxical behavior as the original discrete-strategy games, and it is these continuous-strategy games that form the starting point for our approach to understanding the evolutionary dynamics of the TD and MEC games.

The continuous-strategy forms of the TD and MEC games are two examples of a large class of continuous-strategy games which have a discontinuous payoff function — other important examples of games from this class include the Bertrand Duopoly game [38] and the War of Attrition game [6]. For such games the role played by errors is potentially important. To be precise, let us consider the class of continuous-strategy games with payoff function given by 
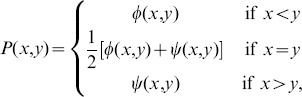
(3)where 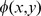 and 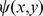 are affine functions, and the strategies 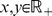. This class of games includes the continuous-strategy variants of both the TD and MEC games. Errors in the observation of an opponents strategy or in the implementation of ones own strategy will result in the expected payoff to an 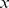-strategist against a 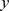-strategist in such a game being given by a function of the form 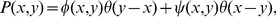
(4)where 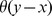 defines the probability that in an interaction between an 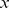-strategist and a 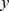-strategist errors lead to the 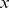-strategist receiving the payoff 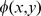, and 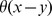 defines the probability that in such an interaction errors result in the 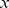-strategist receiving the payoff 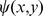. We observe that the probability functions 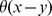 and 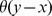 are necessarily complementary in the sense that 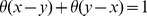, from which it follows that 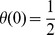. The probability functions 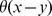 and 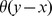 are determined by the statistical distribution of errors in the game. Here, for simplicity, we shall assume that the function 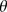 is *smooth*. Thus, the expected payoff (4) defines a *smoothing* of the original discontinuous payoff function (3). We shall often refer to the function 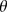 as the smoothing function. In the limit in which the smoothing function tends to the Heaviside step function the expected payoff (4) approaches the payoff (3).

Since the purpose of this paper is to study the evolutionary behavior of the TD and MEC games it is necessary to define a suitable evolutionary dynamics for the class of games that we are considering. Since the effect of errors in the game is to give an expected payoff which is a smooth function, the simplest choice of dynamics is the standard deterministic adaptive dynamics [39]–[41] of the smoothed payoff function. In our approach the effects of errors in the game is encoded in the smoothing function 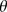. Here we study the evolutionary dynamics of the smoothed versions of the TD and MEC game, for an arbitrary smoothing function, and show that the results obtained are consistent with those found empirically.

The approach to studying the evolutionary dynamics of the TD and MEC games that we follow in this paper has some advantages compared to stochastic learning models [17], [20], [28] or stochastic evolutionary dynamics [24]. In the case of stochastic learning models, the evolutionary dynamics is governed by the Fokker-Planck equation, which is a nonlinear partial differential equation that cannot be solved analytically [17], [20], [28]. The equilibrium solutions of the evolutionary dynamics are given by the solutions to a suitable differential equation. However, it is difficult to determine analytically whether or not the equilibrium solutions are stable. In fact, the equilibrium solutions to the stochastic learning models [17], [20], [28] for both the TD and MEC games have not been shown to be stable, and thus, it is unclear whether or not they are attractors of the evolutionary dynamics.

Stochastic evolutionary dynamics [24] represents an interesting alternative approach to understanding the dynamics of many evolutionary processes. In this case, the evolutionary dynamics is governed by a stochastic process, and the theory is mathematically well-developed. However, there are certain restrictions that apply to the theory. Perhaps the most important is that analytic results can only be obtained in the limit that the selection strength tends to zero. This limit corresponds to the assumption that the contribution to the total payoff that comes from the game interactions is very small. It is not clear that this assumption is realistic when it comes to understanding the behavior of the TD and MEC games. If the assumption of weak selection is not made then it is impossible to obtain analytic results, although numerical simulations can yield results for stronger selection strengths. It is also worth noting that while the stochastic process underlying stochastic evolutionary dynamics is mathematically well-developed, it is rather subtle, and this can present a challenge when applying the method to new problems. In fact, stochastic evolutionary dynamics has not yet been applied to study the MEC game, although we conjecture that such an application will yield results consistent with the behavior observed in the game.

A key advantage of the method that we propose here is that the evolutionary dynamics is much easier to study than for either stochastic learning models [17], [20], [28] or stochastic evolutionary dynamics [24]. In particular, it is straightforward to completely determine the evolutionary attractors for both the TD and MEC games. A second advantage of our method is that it applies directly to a wide variety of continuous-strategy games with discontinuous payoff functions, including the Bertrand Duopoly model [38] and the War of Attrition game [6]. In certain cases, which are considered at greater length in the Discussion, it can be shown using our methods that complex evolutionary dynamics, such as evolutionary branching, can occur in such games.

The rest of the paper is organized as follows. In the Models section, we define continuous-strategy versions of the TD and MEC games, and also introduce the key notion of smoothed versions of these games. In the Analysis section, we analyze the evolution of strategies in the smoothed games in randomly-interacting populations using adaptive dynamics, and in addition formulate an agent-based model of the evolutionary dynamics of these games in populations with structures described by an arbitrary graph (i.e., network). In the Results section, we present the results of simulations using this agent-based model for the evolutionary dynamics of the smoothed TD and MEC games, both in well-mixed populations and in populations described by complex networks. Finally, in Discussion section, we provide a brief discussion of our work and draw some conclusions.

## Models

The strategies in the TD and the MEC games are the claim levels and the effort levels, respectively. Typically these games are taken to have a discrete set of strategies. However, it is in many ways more natural to view the claim levels and effort levels in the two games as being continuously variable, and thus to consider variants of these games defined for continuous strategies. The continuous forms of the TD and MEC games are examples of a broad class of continuous-strategy 2-person games with payoff functions given by 
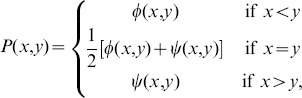
(5)for affine functions 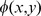 and 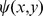, and strategies 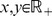. We may write 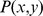 more succinctly with the aid of the Heaviside step function 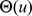


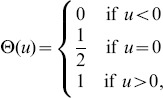
(6)as 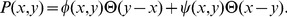
(7)Games of this form have discontinuous payoff functions. Such a discontinuous payoff function is only possible in an idealized world free from all errors. In reality, errors in the perception and implementation of actions in the game will have the effect of replacing the discontinuous payoff function with a smoothed approximation, representing the expected payoff. We now define such a variant of the game in which the discontinuity in the payoff function is removed by a smoothing procedure. To accomplish this we introduce a 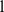-parameter family of smoothing functions 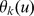. The functions 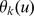 are assumed to be smooth, non-decreasing functions of 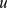, with 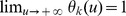, 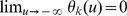, and 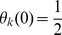. Furthermore, we assume that 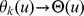 as 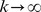. We will refer to the parameter 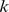 as the smoothing parameter.

To obtain the smoothed version of the game defined by (7) we simply replace 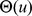 in the payoff function with its smooth approximation 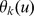. Thus, the payoff function of the smoothed game is given by 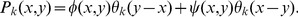
(8)We note that for sufficiently large values of 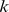 the smoothed game approximates the original game arbitrarily well.

A convenient 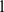-parameter family of smoothing functions is given by 
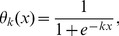
(9)and we shall use this family when explicit smoothing functions are required.

### Traveler's Dilemma Game

The claims made by individuals in the TD game represent their strategies. If 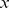 and 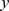 denote the strategies used by two individuals playing the continuous version of the game, and if 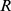 denotes the reward/punishment parameter (where 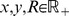), then the payoff to the 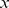-strategist is given by 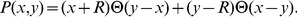
(10)We now define a variant of the TD game defined by (10), in which the discontinuity in the payoff function is removed by the smoothing procedure described above. To obtain the smoothed TD game we simply replace 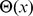 in the payoff function with its smooth approximation 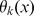. We therefore have that the payoff function for the smoothed TD game is given by 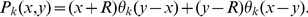
(11)We shall assume, without loss of generality, that the strategy space in the smoothed TD game is the interval 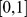, and also that the reward/punishment parameter 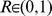. With the payoff function defined by (11), the smoothed TD game represents a natural variant of the original TD game.

### Minimum Effort Coordination Game

In the MEC game, the effort levels of the individuals represent their strategies. In the continuous version of the MEC game, if the strategies of two individuals playing the game are 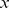 and 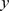 (where 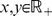) and 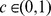 is the effort cost, then the payoff 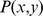 to the 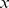-strategist is given by 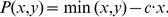
(12)Using (12) allows us to write the payoff function for the MEC game as 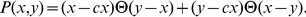
(13)Without loss of generality we can take the strategy space to be the unit interval (i.e. 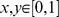). Every strategy pair 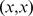 is a Nash equilibrium in this game. The social dilemma embodied in this continuous-strategy game is clearly the same as for the original discrete MEC game: at any equilibrium both players obtain a payoff of 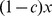, thus all equilibria with the sole exception of the strategy pair 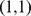 are Hicks inefficient.

To obtain the smoothed MEC game we again replace 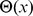 in the payoff function (13) with its smooth approximation 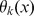. The payoff function of the smoothed MEC game is therefore given by 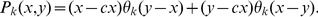
(14)With the payoff function defined by (14), the smoothed MEC game represents a natural variant of the original MEC game.

### Analysis

The dynamics of the smoothed TD and MEC games as formulated in the previous section can be analyzed in a well-mixed population using the deterministic framework of adaptive dynamics [11], [39]–[41]. Consider a monomorphic population in which every individual adopts the same strategy, 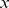. It follows from replicator dynamics that the growth rate of a rare mutant strategy, 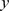, in the resident 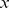 population is 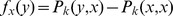, where 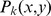 is the payoff to an 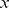-strategist interacting with a 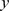-strategist. The quantity 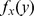 is referred to as the invasion fitness. The evolution of the strategy 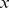 is then governed by the selection gradient 
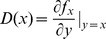
, and the adaptive dynamics of 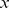 is determined by the differential equation 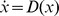.

Equilibrium points of the adaptive dynamics are called singular strategies and are solutions of 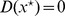. If no such solutions exist, then the strategy 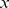 monotonically increases or decreases under evolution, depending on the sign of 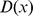. If 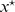 exists, it is convergent stable and, hence an attractor for the adaptive dynamics, if 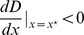. If this equality is reversed, 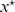 is a repeller.

Initially, the population will converge to a convergent stable singular point 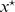, but its subsequent evolutionary fate depends on whether 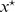 is a maximum or minimum of the invasion fitness 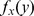. If 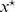 is a maximum, i.e., if 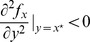, then 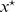 is an evolutionarily stable strategy (ESS), representing an evolutionary end state in which all individuals adopt strategy 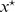. If, however, 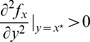, then a population of 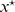-strategists can be invaded by mutant strategies on either side of 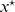. In this case the population undergoes evolutionary branching and splits into two distinct and diverging clusters of strategies.

The adaptive dynamics of smoothed games with payoff function defined by (8) may be analyzed as follows. The invasion fitness is given by 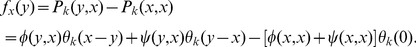
(15)Thus, the selection gradient 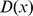 is given by 
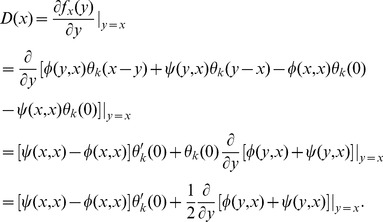
(16)The adaptive dynamics of such a game is therefore determined by the differential equation 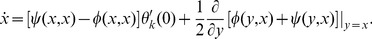
(17)The existence of singular strategies 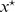 in games of this form, and the particular characteristics of any such 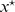, depend on 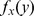 and 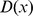, and thus ultimately on the specific functions 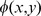 and 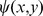. We shall now apply these results to the TD and MEC games.

### Adaptive Dynamics of the Traveler's Dilemma Game

Let us first analyze the TD game with the payoff function given by (11). Consider a monomorphic population of 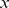 strategists, i.e., a population in which every individual claims amount 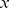. It follows from (2) and (11) that for the TD game 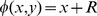 and 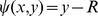. Thus, the selection gradient 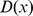 is given by 
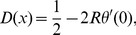
(18)and the adaptive dynamics of 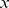 is consequently determined by 
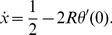
(19)Since 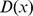 does not depend on 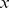, there are no singular strategies, and thus there is no possibility of exotic evolutionary outcomes, such as evolutionary branching. The evolutionary dynamics of an initial strategy 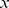 is determined by the sign of 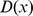. If 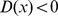 (i.e., if 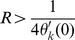), then 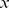 will evolve to 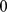. If on the other hand, 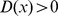 (i.e., 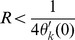) then 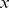 will evolve to 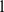. Thus, this adaptive dynamics analysis implies that the players of the smoothed TD game will evolve to make low claims if 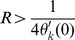, and, conversely, evolve to make high claims if 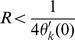. We note that for the 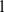-parameter family of smoothing functions defined by (9), this criterion takes the following form: claims will evolve to low levels if 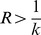, and evolve to high levels if 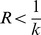.

### Adaptive Dynamics of the Minimum Effort Coordination Game

We next turn to the MEC game where the payoff function is given by (14). Consider a monomorphic population of 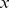 strategists, i.e., a population in which every individual puts in 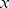 amount of effort. It follows from (2) and (13) that for the MEC game 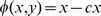 and 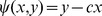. Thus, the selection gradient 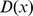 is given by 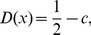
(20)and the adaptive dynamics of 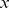 is therefore determined by 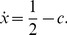
(21)Again, since 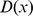 does not depend on 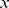, there are no singular strategies. Also, rather remarkably, the adaptive dynamics of 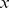 is independent of the smoothing function 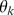. The evolution of an initial strategy 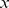 is once again determined by the sign of 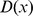. If 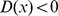 (i.e., if 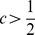) then 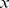 will evolve to 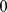, and if 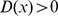 (i.e., if 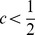) then 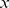 will evolve to 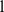. Therefore, this adaptive dynamics analysis implies that the players' strategies in the smoothed MEC game will evolve to low efforts if the effort cost 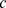 is greater than 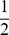, and to high efforts if 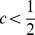. Since the adaptive dynamics of the smoothed MEC game is independent of the smoothing function, these results hold for any smoothing of the game.

We note that the behavior predicted by adaptive dynamics for the smoothed TD and MEC games is in accord with that observed for the TD and MEC games in experiments.

### Agent-Based Simulations

In this section, we define a stochastic agent-based model which allows the evolutionary dynamics of the TD and MEC games to be studied both for random interactions between members of the population and for more complex interaction patterns in the population. The evolutionary dynamics of simple social dilemmas, such as the Prisoner's Dilemma and the Snowdrift game, have been well-studied for populations with a variety of complex interaction patterns [42]–[50].

Consider a population consisting of 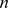 individuals, labeled 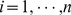. Since we wish to allow the possibility of complex population structures, we identify the population with the set of vertices in a graph 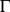. The structure of 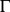 determines which individuals in the population can interact. Strictly speaking, two graphs are required to specify the evolutionary dynamics: an interaction graph, 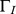, specifies that two individuals in the population can interact by playing the game only if they are adjacent in 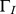, and an updating graph, 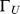, specifies that an individual in the population can update its strategy by comparing its state to the states only of those individuals adjacent to it in 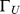. Here, for simplicity, we shall assume that the interaction and updating graphs are the same i.e., 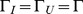. Given an individual 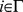, the set of neighbors of 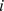 (i.e., the set of individuals adjacent to 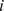 in 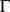) will be denoted by 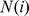.

The agent-based model is defined as a stochastic process on 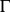. Let us fix either the TD or the MEC game as the game under consideration. We begin with a monomorphic population, i.e., each individual in the population starts out with the same initial strategy randomly picked from a uniform distribution. At each time step 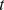, we carry out a round of asynchronous interactions followed by a round of asynchronous updates. Each of these rounds involves sampling the population with replacement.

During an interaction step, we randomly pick an individual 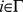 and an individual 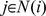, and let the two individuals play the game against each other. If 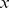 and 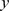 denote the strategies of 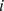 and 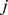, respectively, then the payoff 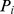 received by the focal individual 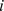 is given by either equation (11) or (14), depending on the game under consideration. This procedure is repeated 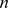 times.

During an update step, we randomly pick an individual 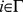 and an individual 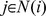. If 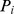 and 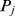 denote the payoffs of 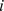 and 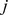, respectively, then with probability 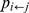 given by 
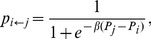
(22)the focal individual 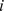 will inherit 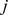
's strategy. This update rule is often referred to as the Fermi rule. The parameter 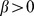 is the “selection strength'' of the update rule. The update procedure is repeated 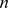 times.

Mutations are incorporated in the update procedure in the following way: when according to the update rule (22) 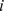
's strategy would be replaced by 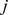
's, then with probability 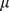, 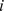
's strategy is instead replaced by a strategy picked randomly from a normal distribution with mean equal to 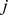
's strategy and standard deviation 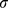. Carrying out 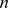 interaction steps followed by 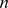 update steps constitutes a single generation of the evolutionary dynamics.

We note here that the results of our agent-based simulations (described in detail in the next section) are robust to variations in the update rule. For example, in addition to employing the Fermi update rule (22), we have also simulated the agent-based model using the *replicator* update rule, in which the probability 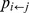 that the focal individual 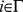 inherits individual 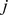
's strategy (with 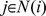) is given by 
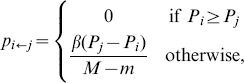
(23)where 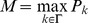, and 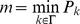. We find that the evolutionary dynamics of the smoothed TD and MEC games is the same irrespective of which of these update rules we employ. The results presented in the next section on the evolutionary dynamics of the TD and MEC games arise from simulations using the Fermi update rule (22).

## Results

In this section we present the results of agent-based simulations for the TD and MEC games. For both the smoothed TD and MEC games the agent-based model described in the previous section was simulated (using the Fermi update rule (22) and the smoothing function (9)) on the following graphs (see, for example, [51]–[54].): a complete graph (which models a randomly-interacting population); a random regular graph of degree 10; a scale-free graph with mean degree 10; and two-dimensional lattice graphs with 4 and 8 neighbors, respectively, and periodic boundary conditions. The games were simulated for 20000 generations. The parameter values used for the simulations were: population size, 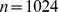 for the lattice graphs and 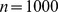 for the other graphs; mutation rate, 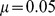; standard deviation for mutations, 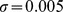; smoothing parameter, 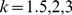 for the TD game and 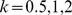 for the MEC game; and selection strength, 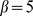.

### Traveler's Dilemma Game

**Figure 2:**
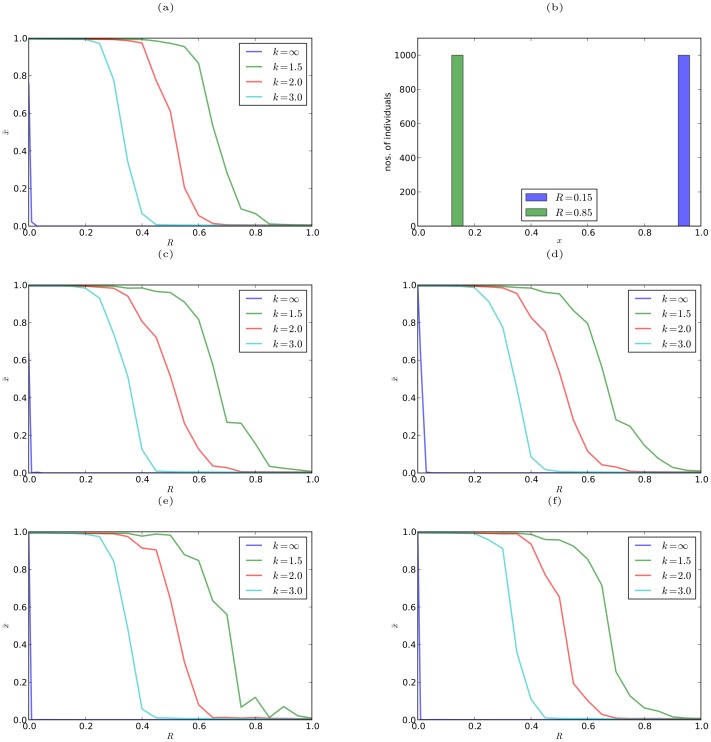
Results from simulating the TD game. (a)(c)(d)(e)(f) Average claims 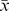 in the smoothed TD game over the last 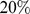 of 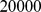 generations versus the reward/punishment parameter 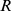 for different values of the smoothing parameter 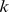, on a complete graph (a), a random regular graph with degree 10 (c), a scale-free graph with mean degree 10 (d), 2D lattice graph with 4 neighbors (e), and 2D lattice graph with 8 neighbors (f). Parameter values: 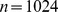 for lattice networks and 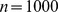 for other networks, 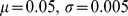, and 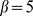. (b) Number of individuals versus their claims 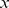, when 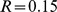 and 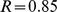, on a complete graph with parameter values: 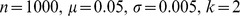, and 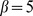.

Figure 2 shows the variation of the average claims 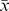 made by individuals over the last 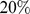 of 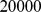 generations with the reward/punishment parameter 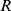, for different values of the smoothing parameter 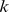, on the following graphs: (a) a complete graph, (c) a random regular graph, (d) a scale-free graph, (e) a 2D lattice graph with 4 neighbors, and (f) a 2D lattice graph with 8 neighbors. The 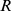 value was varied from 0 to 1 in steps of 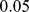, and each data point was obtained from an average of 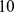 runs of the model. It is apparent from these results that, for each value of 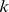, the claims are high when 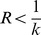 and low when 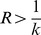, exactly as predicted by the adaptive dynamics analysis. This behavior is in good qualitative agreement with the results obtained for the TD game in experiments. Furthermore, these simulation results suggest that network structure has very little effect on the evolutionary dynamics of the game.

Figure 2(b) shows the variation in the number of individuals with the claims 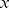 they make, when 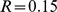 and 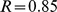, on a complete graph, with 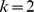. Individuals make higher claims when 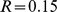, as indicated by the blue bars, and make lower claims when 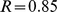, as indicated by the green bars. This result is in good agreement with the empirical results shown in Figure 1(a).

We have also simulated the game using the discontinuous form of the payoff function (10), and the results are shown in blue (labeled 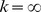) in panels (a), (c), (d), (e), and (f) of Figure 2. In this case, the claims are consistently low for all values of 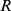. This result, which is consistent with the prediction of the adaptive dynamics analysis of the smoothed TD game in the limit 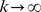, is in agreement with the prediction of classical game theory for the original TD game. Thus, for the TD game studied here, the smoothing of the payoff function is necessary to explain the empirically observed behavior.

### Minimum Effort Coordination Game

**Figure 3:**
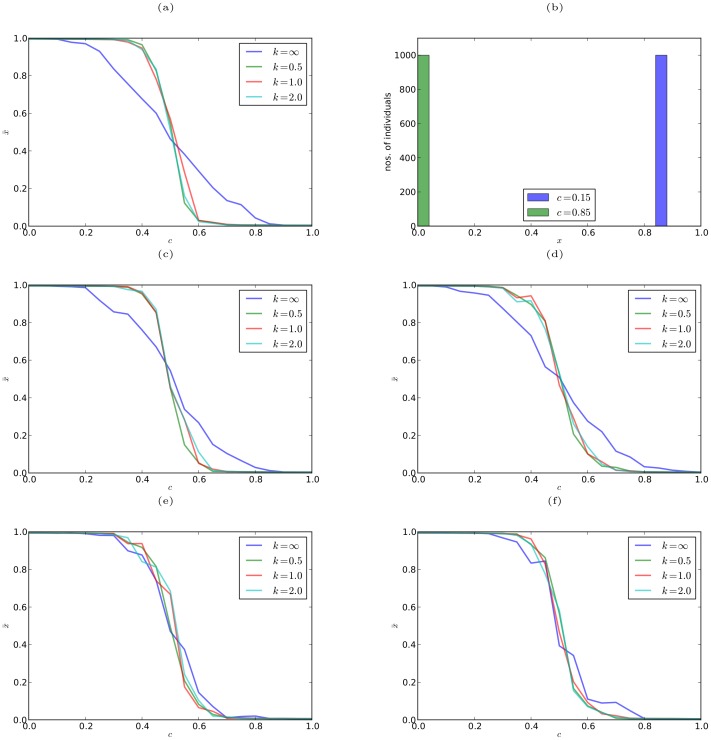
Results from simulating the MEC game. (a)(c)(d)(e)(f) Average effort levels 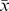 in the smoothed MEC game over the last 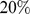 of 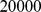 generations versus the effort cost parameter 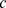 for different values of the smoothing parameter 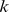, on a complete graph (a), a random regular graph with degree 10 (c), a scale-free graph with mean degree 10 (d), 2D lattice graph with 4 neighbors (e), and 2D lattice graph with 8 neighbors (f). Parameter values: 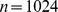 for lattice graphs and 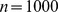 for other graphs, 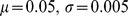, and 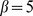. (b) Number of individuals versus their effort levels 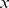, when 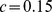 and 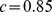, on a complete graph with parameter values: 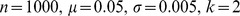, and 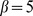.

Figure 3 shows the variation of the average effort levels 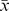 of the individuals over the last 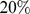 of 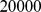 generations with the effort cost parameter 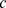, for various values of the smoothing parameter 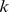, on the following graphs: (a) a complete graph, (c) a random regular graph, (d) a scale-free graph, (e) a 2D lattice graph with 4 neighbors, and (f) a 2D lattice graph with 8 neighbors. The 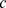 value was varied from 0 to 1 in steps of 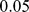, and each data point was obtained from an average over 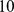 runs of the model. It is clear from these results that, for each value of 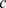, the effort levels are high when 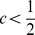 and low when 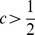, exactly as suggested by the adaptive dynamics analysis. These results are in good qualitative agreement with the empirically observed behavior in experiments. The results of these agent-based simulations clearly show the independence of the evolutionary dynamics of the smoothed MEC game on the smoothing function (i.e., the independence on 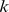), which is predicted by the adaptive dynamics analysis. These results also suggest that the effect of network structure on the dynamics of the smoothed MEC game is negligible.

Figure 3(b) shows the variation in the number of individuals with their effort levels 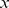, when 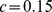 and 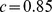, on a complete network, with 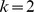. Individuals expend more effort when 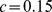, as indicated by the blue bars, and expend lower effort when 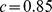, as indicated by the green bars. This result is in good agreement with the empirical results shown in Figure 1(b).

We also simulated the model using the discontinuous form of the payoff function (13), and the result is shown in blue (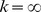) in plots (a), (c), (d), (e), and (f) of Figure 3. In this case, there is still a transition from high to low effort levels as the effort cost increases, however, it is typically not as sharp as for the smoothed game (with any value of 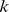). An intriguing exception to this pattern is found in the case of two-dimensional lattice graphs, for which the evolutionary dynamics of the game with discontinuous payoff function is essentially identical to that of the smoothed game (for any value of the smoothing parameter). We tentatively conjecture that the feature of lattice graphs which is responsible for this effect is that they possess non-trivial clustering coefficients ([51]–[54]) — in contrast to the other graphs we have considered, which have zero clustering. The potential effect of the clustering coefficient on the evolutionary dynamics of the MEC game defined on graphs appears to be an interesting topic for future research.

## Discussion

In this work we have proposed simple and natural continuous-strategy versions of the classical discrete-strategy TD and MEC games. We have modeled these games as continuous games with smooth payoff functions, where the smoothing accounts for the effects of errors in the perception and/or implementation of individuals actions. The smoothed TD and MEC games can be effectively analyzed using adaptive dynamics, which shows that the predicted evolutionary dynamics of these games is in accord with the behavior observed in empirical studies of the TD and MEC games. In addition, we have studied the evolutionary dynamics of the smoothed TD and MEC games using agent-based simulations. These simulations have been performed both for populations of randomly-interacting agents and for populations with more complex interaction patterns, represented by graphs of varying topologies. These simulation results are in agreement both with the analytical adaptive dynamics results, and also with the experimentally observed behavior.

For the smoothed TD game, we find both from the adaptive dynamics analysis and from the agent-based simulations, that claims vary with the reward/punishment parameter 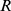 in a fashion that is in excellent agreement with the empirically observed behavior: low values of 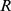 result in high claims and high values of 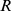 result in low claims. We recover the classical game theory result that claims in the TD game with discontinuous payoff remain low for all values of 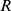 by considering the limit in which the smoothing parameter 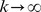. Different interaction patterns among the individuals playing the smoothed TD game, as represented by studying the game on graphs of different topologies, appears to have little effect on the evolutionary dynamics of the game.

We find similarly satisfactory results for the smoothed MEC game. The analysis, both analytical and through simulations, again yields results in good agreement with experiment: high effort levels are found for low effort cost 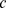 and low effort levels occur for high effort cost. We again find that when the smoothed MEC game is formulated on graphs of differing topology the topological type has no significant effect on the evolutionary dynamics of the game.

The methods introduced in this paper are quite general and can be applied to a wide variety of continuous-strategy games with discontinuous payoff functions. Important examples of other games that can be fruitfully studied using these methods include the Bertrand Duopoly model [38] and the War of Attrition game [6]. Here we will only briefly discuss the application of our methods to these two games — detailed accounts will be given elsewhere.

In the classical Bertrand Duopoly (BD) model [38] one considers the interactions between two firms that produce a homogeneous product. The strategy of each firm is the unit price that they set for their product. It is assumed that customers will buy a quantity 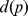 (where 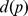 is the demand function) from the firm with the lower price 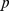, and will buy nothing from the firm with the higher price. If both firms set the same price then it is assumed that demand is split equally between them. It is also assumed that both firms have the same marginal cost 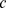. Thus, the payoff obtained by one firm in its interaction with the other is a discontinuous function of the difference between the prices set by the two firms. This discontinuous payoff function can be smoothed in exactly the manner described in this paper to yield a smoothed game. In this case the smoothing function represents the probability that customers buy the product from the firm with the lower price as opposed to the firm with the higher price. In the limit that this probability tends to one, the smoothed game approaches the classical game.

The smoothed BD model can be analyzed using adaptive dynamics just as we have done here for the TD and MEC games. It may be shown for an arbitrary smoothing, that given a linear demand function 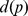 there exists a unique singular strategy 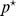 (in the domain of interest), that is strictly greater than the marginal cost 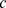. Furthermore, it may also be shown that the singular strategy 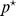 is always both convergent stable and an ESS. Thus, the evolutionary dynamics of the smoothed BD game results in the prices set by both firms converging to the level 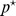, which is strictly greater than the marginal cost. At the evolutionary equilibrium 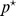 both firms obtain a positive payoff. Thus, the introduction of the smoothed BD model effectively resolves the Bertrand Paradox that occurs in the classical model. Moreover, our method extends to the case of an arbitrary number of firms and sheds light on the results obtained in [55].

The second example that will be mentioned here is the War of Attrition (WoA) game [6]. The classical WoA game is concerned with two individuals who are contesting a resource. In this game each individual chooses a ''display investment,'' which is a continuous variable representing the individual's strategy. The payoff in the classical game is a discontinuous function of the difference in the display investments since it is assumed that the individual with the higher investment obtains the resource, while the lower investor does not, and both pay a cost that is a function of the lower investment value.

This game can be smoothed and studied using adaptive dynamics as we have done in this paper for the TD and MEC games. It may be shown that the evolutionary dynamics depends critically on the form of the cost function. For a linear cost function, there exists a singular strategy that is always both convergent stable and an ESS. However, for quadratic cost functions, there exist singular strategies that are evolutionary branching points. Thus, in the latter case, complex and surprising evolutionary dynamics can occur.

In conclusion, therefore, we have introduced a new method of formulating and analyzing the evolutionary dynamics of a wide class of games, which include the continuous-strategy variants of the TD and MEC games. We have studied the case of the smoothed TD and MEC games in detail and shown that our method provides a means of resolving the paradoxical behavior associated with the original form of the games.
